# Binocular integration and stereopsis in children with television torticollis

**DOI:** 10.1186/s12886-021-01850-5

**Published:** 2021-02-24

**Authors:** Cheng Yang, Wanshu Huang, Ying Cui, Guanrong Zhang, Dongmei Wang, Wenjuan Xie, Mark Wiederhold, Brenda Wiederhold, Hang Chu, Li Yan, Jin Zeng

**Affiliations:** 1 Department of Ophthalmology, Guangdong Eye Institute, Guangdong Provincial People’s Hospital, Guangdong Academy of Medical Sciences, Guangzhou, Guangdong China; 2 LiKaShing Faculty of Medicine, The University of Hong Kong, 6 William MW, Mong Block 21 Sassoon Road, Pokfulam, HongKong SAR China; 3 Information and Statistics Center, Guangdong Provincial People’s Hospital, Guangdong Academy of Medical Sciences, Guangzhou, Guangdong China; 4 Virtual Reality Medical Center, Scripps Memorial Hospital, 9834 Genesee Suite#427, La Jolla, California, USA; 5 National Engineering Research Center for Healthcare Devices, 1307 Guangzhou Avenue Middle, Guangzhou, Guangdong China

**Keywords:** Television torticollis, Binocular integration, Stereopsis, Virtual reality

## Abstract

**Background:**

To observe the characteristics of binocular integration and stereopsis in children with television torticollis.

**Methods:**

A retrospective study was carried out, where data were collected from 25 children with television torticollis as the disease group after refractive error correction and 25 normal children as the control group. A virtual reality system was used to assess and analyze the characteristics of binocular integration by a contrast balance test and binocular stereopsis.

**Results:**

The 25 children in the disease group included 17 males and 8 females with an average age of 7.5 ± 1.9 years old and an average binocular spherical equivalent of − 0.35 ± 1.46D. The 25 children in the control group were also 17 males and 8 females with an average age of 7.3 ± 2.2 years old and the average binocular spherical equivalent of − 0.48 ± 0.93D. No significant differences were found in the horizontal bar contrast balance test between the 2 groups at near and far distances. Near-distance vertical bar contrast balance test was normal in 23 subjects and suppressed in 2 subjects in the control group, while it was normal in 13 subjects and suppressed in 12 subjects in the disease group, which showed a statistically significant difference (*P* = 0.002). Far distance vertical bar contrast balance test was normal in 24 subjects and suppressed in 1 subject in the control group, normal in 7 subjects and suppressed in 18 subjects in the disease group, showing a statistically significant difference (*P* = 0.000). All subjects in the 2 groups showed 100〞 as near distance stereoacuity. At far distance, the mean stereoacuity was 176.00〞 ± 92.56〞 in the control group, and 352.00〞 ± 270.99〞 in the disease group, with a statistically significant difference (*P* = 0.011).

**Conclusion:**

By using virtual reality technology, defects in binocular visual function were found in children whose television torticollis persisted after regular refractive error correction. Television torticollis may be associated with the deficit of binocular integration for vertical bars and far distance stereopsis.

## Background

Anomalous head posture (AHP) is a common condition in children, with an incidence of 1.3% [1]. An AHP can take the form of head tilt, face turn, chin up, chin down or combined [2]. The cause of AHP has been mainly assigned to ocular [2–4], orthopedic [5, 6], and neurologic causes [7, 8].

Television (TV) torticollis refers to an AHP among school age children while they are watching television, which may not be entirely attributed to any of the above causes [9]. It is defined as a condition that the patient’s head and eye positions as well as eye movements are normal, except when the patient is concentrated on watching television where the head involuntarily turns to one side while both eyes look to the contralateral side, without any overt ophthalmological problems [10–13]. There have been some studies on television torticollis, suggesting that it may be related to refractive error, psychological habits, or the incomplete development of visual function [9–15]. In our previous clinical practice, we found that television torticollis still persisted despite refractive error correction in many children. The exact pathogenesis and effective treatment of the disease still need in-depth research to figure out.

In this study, stereopsis and binocular integration assessed by a contrast balance test were compared between children with television torticollis after regular refractive error correction, and age matched normal children. The aim of the study is to explore the binocular visual function defects in television torticollis children using a virtual reality system.

## Methods

### Subject selection

A retrospective study was carried out, and data were collected from children with complaints of torticollis when watching television as defined above, and age matched normal children, who were under treatment in the Ophthalmology Department of The Guangdong Provincial People’s Hospital from 1st January 2016 to 31st December 2017. Assessments were carried out in all children, including: uncorrected visual acuity, best corrected visual acuity (through cycloplegic and subjective refraction), intraocular pressure, slit lamp examination, fundus examination, eye position and ocular movement examination. The degree of astigmatism was converted to spherical equivalent (SE) degree. Examinations of refraction and eye position were done by the same optometrist. Exclusion criteria included: orthopedic or neurologic causes of torticollis, anisometropia (interocular difference in spherical equivalent ≥2.5D), amblyopia, manifest strabismus, latent strabismus, nystagmus, severe ocular infection, organic lesion of the eye, history of ocular surgery, and children who could not understand or cooperate during assessment of stereopsis and contrast balance test. Torticollis patients first underwent refractive error correction with 6 months of regular eyeglass treatment and were recruited into the study if torticollis persisted.

Finally, 25 patients were recruited into the TV torticollis group, and 25 normal children were recruited into the control group (see Table 1 for summary demographics). Written informed consent was obtained from all patients or their guardians before data collection commenced. All study protocols were approved by the Ethics Committee of Guangdong Provincial People’s Hospital and carried out in adherence to the Declaration of Helsinki with regard to ethical principles for research involving human subjects.

**Table 1:** Summary Demographics of Patients in the Study

	Control group	TV torticollis group	*P*-value
Patients ^*a*^	25	25	
Gender ^*a*^ (male: female)	17:8	17:8	
Age ^*b*^	7.3 ± 2.2	7.6 ± 1.9	0.481 ^*c*^
Interocular SE (D)	0.11 ± 0.29	0.01 ± 0.41	0.350 ^*c*^
Mean SE (D)	−0.48 ± 0.93	−0.35 ± 1.46	0.329 ^*c*^
Median columnar (D)	−0.25	−0.375	0.276 ^*d*^

^a^ Numbers of subjects

^b^ Average years of age at first diagnosis

^c^ From the independent t-test

^d^ From the Mann-Whitney rank-sum test

*SE* spherical equivalent; *D* diopters

### Assessment device

The stereopsis and contrast balance test were performed by a virtual reality system, designed and invented by the National Engineering Research Center for Healthcare Devices (license number: Guangdong Machinery Registration 20,142,700,073). The stimulus template in the system was generated by MATLAB, and the stimulus images were displayed on a three dimension (3D) monitor (LGD2343P with a resolution of 1980 × 1080 and a refresh rate of 120HZ). All tests were conducted at a constant room luminance, and all patients wore their spectacle corrections and 3D polarized glasses. The distance was divided into near distance (80 cm from the display) and far distance (3 m from the display). The size of image was changed to an equal proportion in order to get the same image on the subject’s retina without optical parallax at the two viewing distances. All children’s examinations were performed by the same skilled operator and repeated for at least 3 times to get the average data.

### Assessment of binocular integration

Binocular integration was assessed by a contrast balance test based on reference [16]. A grey background of 44 cd/m^2^ was displayed on a 3D monitor, the viewing angle was 38° × 18°, and the size of horizontal and vertical bars were both 0.8° × 0.8°. Three different resolutions of square targets (50 × 50 pixels, 100 × 100 pixels, 200 × 200 pixels) were presented in horizontal and vertical bars respectively. The assessment distance was divided into near distance (80 cm) and far distance (3 m).

**Fig. 1:**
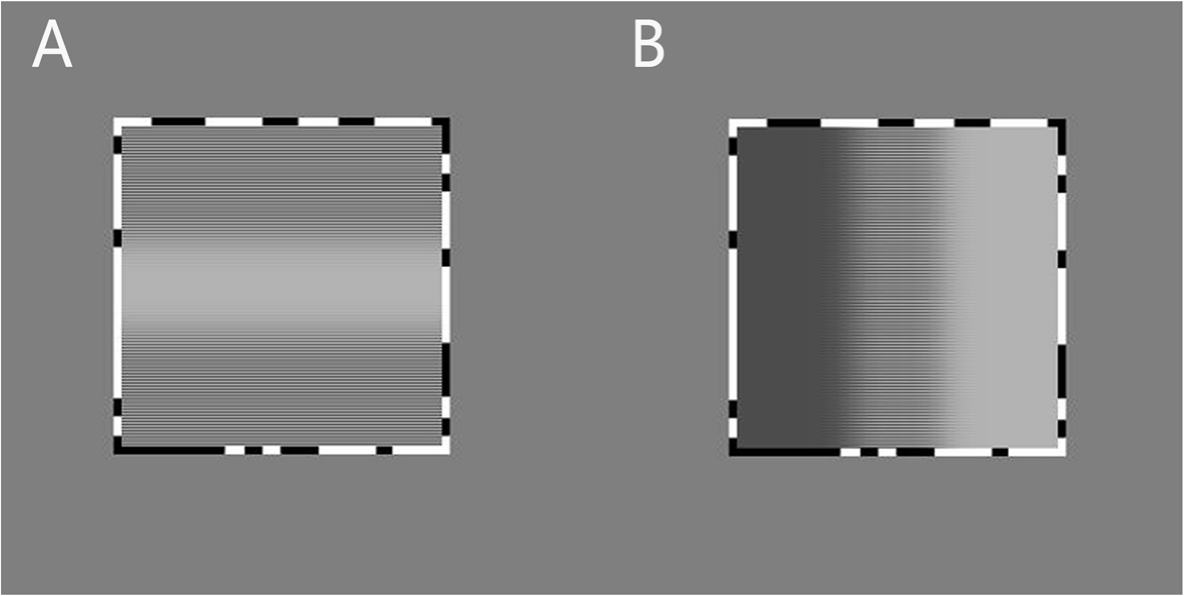
Image of contrast balance test. **a**. Horizontal bar contrast balance test image: normal subjects would see equal amounts of black horizontal bars in upper and lower portions of the image. **b**. Vertical bar contrast balance test image: normal subjects would see equal amounts of black, grey and white vertical bars

The subjects wore 3D polarized dichoptic glasses to view the images on the screen. The two eyes were respectively shown the horizontal (vertical) bars and the horizontal (vertical) bars that differentiate by half a phase. The two images overlapped in the outline but induced partial binocular rivalry through dis-synchronized phases. Participants were then asked to determine whether and how much the areas were white, grey or black. The test results were categorized as normal or suppressed. If the contrast balance test was normal, an equal amount of upper and lower horizontal black bars should be seen in the horizontal bar (Fig. 1-a), or an equal amount of vertical black, grey and white bars would be seen in vertical bar (Fig. 1-b), in all the grades of resolutions respectively. If not so, the contrast balance of the dominant eye would be reduced from 100 to 5%, by a reduction of 5% each time, until the equal amount was seen, the percentage of contrast balance was recorded. We define the percentage of dominant eye lower than 90% as suppressed.

### Assessment of Stereoacuity

A random dot distribution map on a grey background of 125 and a luminance of 44 cd/m^2^ was presented on a 3D monitor. One-thousand two hundred and fifty random dots with a grey background of 250 were distributed in a 5° × 5° square area. Patients viewed a central optotype, an E-target of 3° × 3° in the central portion of the random dot distribution map, with different disparity as 400 〞, 300 〞, 200 〞, and 100〞. The surrounding random dots were used as reference for relative non-parallax [17].

**Fig. 2:**
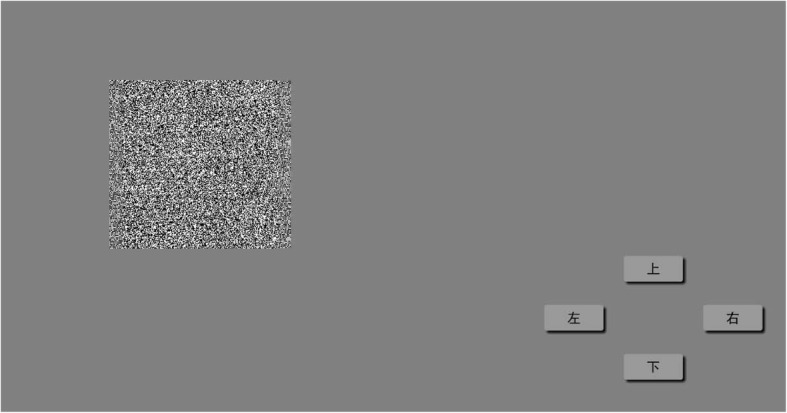
Image of stereoacuity assessment. Patients wearing 3D polarized glasses were asked to look at an optotype “E” on one monitor and used the arrow keys to record their directions

The subject wore 3D polarized glasses and was instructed to determine the direction of the protruding E-target opening shown on the screen by clicking on the corresponding arrow icon using either a mouse or by pressing the corresponding arrow key of the keyboard (Fig. 2). At the beginning stage, the subject viewed a protruding E-target with 400 〞 and was asked to determine the direction of E-target opening twice. If the subject answered correctly both the two times, the protruding E-target with 300 〞 would be shown to the subject, et cetera, until 100〞. If the subject made a wrong answer for one time, the test would go back to the previous higher disparity level. The final result was recorded. The assessment distance was divided into near distance (80 cm) and far distance (3 m). Stereoacuity was recorded as seconds of arc.

### Statistical analysis

Statistical analysis was performed using SPSS Statistics for Windows (ver. 22.0. IBM Corp., Armonk, NY). The age, SE and stereoacuity were presented as mean ± standard deviation. Comparison of the age, interocular SE and mean SE between the two groups was made using the independent *t*-test with a *P*-value < 0.05 being a statistically significant difference. The comparison of columnar degree, contrast balance test and stereopsis between the two groups was made using the Mann-Whitney rank-sum test, with a *P*-value < 0.05 being a statistically significant difference.

## Results

### Demographic of patients

The 25 patients recruited in the TV torticollis group were 15 males and 8 females ranged between 6 and 11 years old with an average age of 7.6 ± 1.9 years, a mean spherical equivalent degree of − 0.35 ± 1.46D and an average interocular difference in spherical equivalent degree of 0.01 ± 0.41D, a median columnar degree of − 0.375D. The 25 children recruited into the control group were also 15 males and 8 females, who ranged between 5 and 12 years old, with an average age of 7.3 ± 2.2 years old. Their mean spherical equivalent degree was − 0.48 ± 0.93D, the average interocular difference in spherical equivalent degree was 0.11 ± 0.29D, and the median columnar degree was − 0.25D. The gender ratio was the same in the two groups. The differences between their ages (*P* = 0.481), mean spherical equivalent degrees (*P* = 0.329), average interocular spherical equivalent degrees (*P* = 0.350) and columnar degrees (*P* = 0.276) were not statistically significant (Table 1).

The Comparison of Near and Far Distance Contrast Balance Test between the Two Groups.

**Fig. 3:**
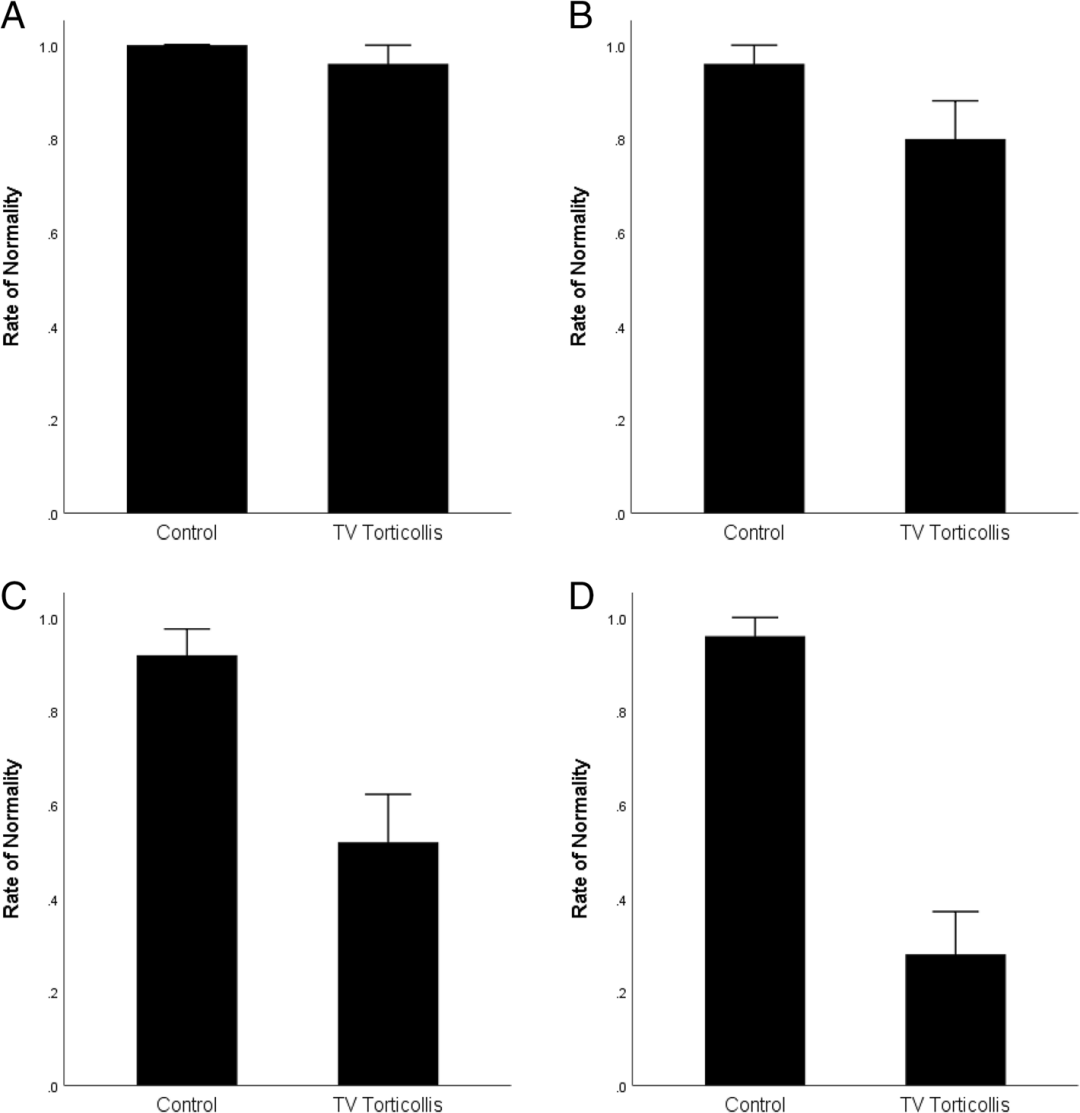
Comparison of near and far distance contrast balance test between the two groups. **a**. Bar graph showing the rates of subjects with normal near distance horizontal bar contrast balance test between the two groups. There was no statistically significant difference between the two groups (*P* = 0.317). **b**. Bar graph showing the rates of subjects with normal far distance horizontal bar contrast balance test between the two groups. There was no statistically significant difference between the two groups (*P* = 0.085). **c**. Bar graph showing the rates of subjects with normal near distance veritical bar contrast balance test between the two groups. The rate of normal subjects was higher in the control group than that in the TV torticollis group (*P* = 0.002). The difference is statically significant. ** Statistically significant difference. **d**. Bar graph showing the rates of patients with normal far distance vertical bar contrast balance test between the two groups. The rate of normal subjects was much higher in the control group than that in the TV torticollis group (*P* = 0.000). ** Statistically significant difference

Near distance horizontal bar contrast balance test: all 25 subjects were normal in the control group and none showed suppression, whereas in the TV torticollis group, 24 were normal but 1 subject showed suppression. There was no statistically significant difference between the two groups (*P* = 0.317) (Fig. 3-a).

Far distance horizontal bar contrast balance test: 24 subjects were normal, and 1 subject showed suppression in the control group, whereas 20 subjects were normal, and 5 subjects showed suppression in the TV torticollis group. There was no statistically significant difference between the two groups (*P* = 0.085) (Fig. 3-b).

Near distance vertical bar contrast balance test: 23 subjects were normal and 2 were suppressed in the control group, whereas only 13 were normal and 12 were suppressed in the TV torticollis group. The difference between the two groups was statistically significant (*P* = 0.002) (Fig. 3-c).

Far distance vertical bar contrast balance test: 24 subjects were normal, and 1 subject showed suppression in the control group, while only 7 subjects were normal and 18 showed suppression in the TV torticollis group. The difference between the two groups was statistically significant (*P* = 0.000) (Fig. 3-d).

The Comparison of Stereoacuity at Near and Far Distance between the Two Groups.

All children in the control group and the TV torticollis group showed 100〞as near distance stereoacuity, and there was no difference between the two groups (*P* = 1.000). At far distance, the mean stereoacuity was 176.00〞 ± 92.56〞 in the control group, and 352.00〞 ± 270.99〞 in the TV torticollis group. The difference between the two groups was statistically significant (*P* = 0.011) (Table 2).

**Table 2:** Comparison of Stereoacuity between the Two Groups

	Control group	TV torticollis group	*P*-value
Near distance stereoacuity ^*a*^	100.00 ± 0.00	100.00 ± 0.00	1.000 ^*b*^
Far distance stereoacuity ^*a*^	176.00 ± 92.56	352.00 ± 270.99	0.011 ^*b*^

^a^ Seconds of arc

^b^ From the Mann-Whitney rank-sum test

## Discussion

In this study, television torticollis was referred to as an AHP appeared only when the patient was focused on watching television, without any overt ophthalmic problems, orthopedic or neurologic diseases. The main finding of our study was the abnormality in far distance stereopsis and vertical bar contrast balance test in the TV torticollis group, compared with that in control group.

Zhang D et al. found that there was an association between fusional vergence dysfunction and the AHP children without obvious refractive errors when watching television [9]. Some studies reported that AHP was associated with refractive errors, including undercorrected myopia, anisomyopia, overcorrected hyperopia, high hyperopia or astigmatism [3, 18–23]. According to the studies, AHP could be partly or totally eliminated when the glasses were worn. During our clinical practice, it is commonly seen that many patients with AHP when watching television have symptoms that persist after regular refractive error correction, and the disease also took place in many patients with physiological astigmatism. This study recruited patients with persistent television torticollis after regular eyeglass treatment for at least 6 months.

Previous studies have found that television torticollis is more common among school-age children 6–12 years old, with a male to female ratio of 3:2 [11–13]. The 25 patients recruited in this study were 17 males and 8 females ranging from 6 to 11 years old, which approximated the age and gender of patients to previous studies.

Zhang et al. [15] believed that the cause of television torticollis might be related to astigmatism and they proposed a hypothesis as follow: the distance between the two focal lines (Sturm interval) is increased in an astigmatism eye. The circle of least diffusion is also increased, causing the retinal image formed to be blurry. When the head is tilted, the inflection of light entering the eye may be affected which reduces or eliminates the Sturm interval, and subsequently reduces the circle of least diffusion, increasing the clarity of the image. In addition, the pupil of an intorted eye is partially blocked by the nasal bridge causing a pinhole effect, which also reduces the Sturm interval. We believe this hypothesis may be a mechanism leading to television torticollis, and we further assume that the tilted head may be related to binocular integration, which means interocular dominance and suppression [16]. It is speculated that children tend to tilt their head to the more “suppressed” eye and look at the monitor by the more “dominant” eye in order to maintain binocularity and see comfortably. Parents verified that their children with torticollis did not switch head sides while watching television at home. During the assessments of binocular visual function in hospital, the patients’ anomalous head posture mainly took the form of head tilt and face turn, sometimes combined with chin up or chin down. We did not find the torticollis children switched head sides either. In our study, binocular integration were measured using a contrast balance test based on previous reference [16]. We explored for the first time, the relationship between television torticollis and binocular integration. The results of this study showed that the control group had better developed contrast balance towards vertical bar than the TV torticollis group, and the two groups demonstrated a similar contrast balance of horizontal bars. These findings implied the assumption that vertical bar contrast balance might be an important indicator for binocular integration in television torticollis children and the disease might be associated with binocular integration, but it still needs in-depth study to be testified. There may be a relationship between the vertical bar contrast balance and the monitor of television, which also needs to be conducted in the future.

There was no statistically difference in stereoacuity between television torticollis children and normal children stated by other researchers [9, 24, 25]. This is consistent with the finding in our study that all the children in TV torticollis group had normal near distance stereoacuity. However, we determine that the distance of watching television is usually from a far distance (2–3 m away). In this study, children in the control group had better far distance stereoacuity than patients in the TV torticollis group (*P* = 0.011), which implied that the defect in far distance stereoacuity may be associated with television torticollis. Studies have shown that the far distance zero-order stereopsis represents the fine stereopsis dominated by the visual cortex parvocellular pathways [26]. It is possible that the poor development of the parvocellular pathways in the visual cortex may also be associated with television torticollis. In previous studies on stereoacuity of television torticollis, the assessments were generally limited to near distance only, making the approach difficult to uncover defects in binocular visual function in the children.

There are some limitations in this study such as small sample size and the lack of a prospective study. Also, the resolution of the 3D monitor was not high enough to show disparities lower than 100 arcsec in the stereoacuity test. Based on the results of this study, we intend to perform binocular visual function test with an upgraded monitor and investigate the relationship between dominant eye and the form of anomalous head positions on a larger sample of children with television torticollis, in order to develop a possible individualized binocular visual function training program and observe its therapeutic effect.

## Conclusion

In conclusion, by using virtual reality technology, defects in binocular visual function were found in children whose television torticollis persisted after regular refractive error correction. In particular, television torticollis may be associated with the deficit of far distance stereopsis and binocular integration for vertical bars, which may imply a suboptimal development of the corresponding areas in the visual cortex.

## Data Availability

The datasets used and/or analyzed during the current study are available from the corresponding author on reasonable request.

## Abbreviations

TV: Television
3D: Three dimension
